# Multiparametric MRI in Prostate Cancer

**DOI:** 10.1155/2014/296810

**Published:** 2014-12-01

**Authors:** Tarık Esen, Barıs Turkbey, Anup Patel, Jurgen Futterer

**Affiliations:** ^1^Department of Urology, School of Medicine, Koc University, 34450 Istanbul, Turkey; ^2^Molecular Imaging Program, NCI, NIH, Bethesda, MD 20892, USA; ^3^Royal London Hospital, Barts Health NHS Trust, London E1 1BB, UK; ^4^Department of Radiology, Radboud University Nijmegen Medical Centre, 6500 HB Nijmegen, The Netherlands

Prostate cancer (PCa) is the second most common cancer in men and an estimated 1.1 million men worldwide were diagnosed with prostate cancer in 2012 with almost 70% of the cases occurring in more developed regions [1]. With an estimated 307.00 deaths in 2012, prostate cancer is the fifth leading cause of death from cancer in men [1]. In patients with elevated or rising prostate-specific antigen (PSA) and/or abnormal digital rectal examination (DRE), random systematic transrectal ultrasound (TRUS) guided prostate biopsy is the most commonly used technique to establish PCa diagnosis. However, PSA alone has a low specificity for PCa detection [2] and several modifications that may improve the specificity of PSA in PCa diagnosis have been described such as age-specific reference ranges, free-to-total PSA ratio, PSA velocity, PSA density, PSA transition zone density, PSA molecular forms (PHI (prostate health index) score (total PSA, free PSA, and p2PSA) and the 4Kscore, which measures blood plasma levels of four different prostate-derived kallikrein proteins: total PSA, free PSA, intact PSA, and human kallikrein 2 (hK2)), and numerous other novel biomarkers (PCA3, TMPRSS2-ERG Gene fusion, etc.).

Random systematic prostate sampling with TRUS guided biopsy also has its inherent limitations. First of all, clinically insignificant cancers are often identified by chance and affect survival data due to lead and length time bias from overdetection and overtreatment of indolent disease [3]. Secondly, systematic biopsies through random sampling error may lead to incorrect risk stratification and may perform poorly at documenting the exact extent and heterogeneity of the disease [4]. Lastly, undersampling, particularly, when prostate volume is taken into account, occurs in up to 30% of cases with clinically significant tumors being missed (false-negativity) on initial random systematic biopsy [5]. Efforts to overcome these sampling errors include performing multiple repeat random biopsies or increasing the core number during random or systematic template-guided transperineal saturation biopsies. However, this approach results in a marginal increase in the overall detection rate without increasing the rate of significant cancer detection [6], while increasing cost and morbidity significantly.

Imaging has always been problematic in respect to PCa diagnosis and has been essentially limited to biplanar transrectal ultrasound and its modifications. Technological ultrasound developments with contrast enhancement and 3D reconfiguration have been underutilized by the urological community as a whole. Isotopic efforts with FDG and 11-choline PET scans have also disappointed in terms of diagnostic yield of prostatic disease. Recently, with the advent of better technology and different sequences, magnetic resonance imaging (MRI) has shown to be able to play a significant role in this respect. Numerous studies have now indicated that multiparametric (Mp) prostate MRI at 3 Tesla, including anatomical and functional sequences, enables accurate PCa detection and local staging with reasonable sensitivity and specificity [7]. Given that, Mp-MRI might be useful in image-guided therapy such as focal therapy, as well as in whole gland therapy such as radical prostatectomy or modern techniques of external beam and interstitial radiotherapy.

Sensitivity for detecting larger (>5 mm) and more aggressive (Gleason score > 7) tumors is better for Mp-MRI, indicating that it may preferentially detect clinically relevant tumors [8]. However, in most studies histological correlations were done using targeted or systematic ultrasound guided biopsy samples and this does not represent a perfect coregistration. Blinded multicenter studies that validate Mp-MRI findings with biopsy and then whole-mount prostatectomy histopathology are lacking. Only until these are done can we determine Mp-MRI's performance for detecting clinically significant cancers in different prostate gland locations (central zone, apical region, anterior peripheral zone, etc.).

In an effort to standardize the reporting of Mp-MRI findings and to reduce subjectivity of image interpretation, the PI-RADS (prostate imaging and reporting archiving data system) classification has been developed. In this scoring system every parameter (T2WI, DWI, DCE-MRI, and MRSI) is scored on a five-point scale. Additionally, each lesion is given an overall score, to predict its chance of being a clinically significant cancer [9]. Nevertheless, given the fact that the minimum and optimum requirements regarding general MRI components and MRI sequence parameters and techniques are not universally agreed upon, there remains the “Achilles Heel” of considerable interobserver variation. As a result, more prospective studies have to be executed in order to validate the accuracy and interobserver variability of multiparametric prostate imaging.

Mp-MRI represents a potential tool to address the limitations of contemporary random systematic biopsy related to undersampling and false-negativity. Among biopsy-naive men, it increases the frequency of significant cancer detection to 50% in low-risk (PSA < 10 ng/mL, normal DRE) and 71% in high-risk (PSA > 10 ng/mL, abnormal DRE) patients [10]. In low-risk men, the negative predictive value of a combination of low level of suspicion based on MRI with prostate volume parameters was nearly 98% and this might be useful to avoid biopsy for those who will not need immediate active treatment if diagnosed with prostate cancer [10] but must compete on cost terms with novel blood-urine tests. Among men with a previous negative TRUS guided random biopsy but persistent clinical suspicion, 72% to 87% of cancers detected by MRI guidance were clinically significant [11].

Among active surveillance candidates, repeat biopsy using MRI targeting demonstrates high sensitivity in confirming low-risk disease in low suspicion score lesions and risk upgrading in highly suspicious lesions [12].

Techniques of MRI targeted biopsy include visual cognitive transrectal ultrasound guided biopsy, software coregistered magnetic resonance imaging-ultrasound, transrectal ultrasound guided biopsy, and in-bore magnetic resonance imaging guided biopsy. While many studies have compared targeted biopsy to systematic biopsy, few have evaluated the cancer detection rates across different targeted techniques. Fusion targeting seems to improve accuracy for smaller MRI detected lesions as well as high-grade cancer (Gleason score > 7) [13]. However, the optimal method for MR targeted biopsy remains in evolutionary flux and has yet to be established.

Mp-MRI is also a feasible and very useful tool in detecting local recurrence in patients who have undergone radical prostatectomy (RP) or radiotherapy. Concerning post-RP local recurrences, DCE-MRI seems to be the most reliable technique for detection, though DWI can be proposed as a reliable alternative [14]. For predicting locally recurrent PCa after radiation therapy, the use of combined T2WI and DWI showed a better diagnostic performance compared to T2WI alone [15].

Is Mp-MRI ready for prime time? Well, this question remains open. Currently, the use of Mp-MRI as a “screening” tool for men referred with abnormal PSA for biopsy decision-making cannot be a general recommendation. However, it will undoubtedly have a place alongside novel biomarkers in diagnostic and surveillance strategies, in repeat biopsy settings, and perhaps in surgical planning for nonmetastatic bulky local disease as part of a planned multimodality treatment regimen.

We hope that the readers of this special issue with 11 manuscripts which explores different aspects of Mp-MRI for diagnosing and treating PCa in its different forms will find some answers for different clinical settings. We are confident that this very timely compilation will be a major reference for many of us interested in prostate cancer.
